# Socioeconomic determinants of stay-at-home policies during the first COVID-19 wave

**DOI:** 10.3389/fpubh.2023.1193100

**Published:** 2023-07-05

**Authors:** Pablo Valgañón, Unai Lería, David Soriano-Paños, Jesús Gómez-Gardeñes

**Affiliations:** ^1^Department of Condensed Matter Physics, University of Zaragoza, Zaragoza, Spain; ^2^GOTHAM Lab - Institute for Biocomputation and Physics of Complex Systems (BIFI), University of Zaragoza, Zaragoza, Spain; ^3^Institute Gulbenkian of Science (IGC), Oeiras, Portugal

**Keywords:** COVID-19, epidemic modeling, Bayesian inference, compartmental models, non-pharmaceutical containment policies

## Abstract

**Introduction:**

The COVID-19 pandemic has had a significant impact on public health and social systems worldwide. This study aims to evaluate the efficacy of various policies and restrictions implemented by different countries to control the spread of the virus.

**Methods:**

To achieve this objective, a compartmental model is used to quantify the “social permeability” of a population, which reflects the inability of individuals to remain in confinement and continue social mixing allowing the spread of the virus. The model is calibrated to fit and recreate the dynamics of the epidemic spreading of 42 countries, mainly taking into account reported deaths and mobility across the populations.

**Results:**

The results indicate that low-income countries have a harder time slowing the advance of the pandemic, even if the virus did not initially propagate as fast as in wealthier countries, showing the disparities between countries in their ability to mitigate the spread of the disease and its impact on vulnerable populations.

**Discussion:**

This research contributes to a better understanding of the socioeconomic and environmental factors that affect the spread of the virus and the need for equitable policy measures to address the disparities in the global response to the pandemic.

## 1. Introduction

The COVID-19 disease, caused by the novel coronavirus SARS-CoV-2, is a highly contagious respiratory illness that was first reported in Wuhan, China in December 2019 (1). It was declared a pandemic by the World Health Organization on March 11th 2020 and by that time, the disease had spread globally, resulting in an international public health crisis that impacted all aspects of life for those affected. As it continued to spread, countries worldwide implemented a range of strict measures to contain the virus with varying degrees of success, including lockdowns, travel restrictions, and social distancing measures (2). Since the early stages of the pandemic, experts have studied the uneven spatial spread of the virus, which is associated with socioeconomic and environmental factors (3, 4). These studies revealed that minorities, low-income areas, and vulnerable populations (5, 6) have been disproportionately affected by the situation, exacerbating existing inequalities.

Numerous studies have examined the factors that contribute to the reproduction number or the speed at which a disease propagates in a population (7). Most of these studies have found positive correlations between the transmissibility of a pathogen and factors such as population density, income inequality, urban areas, and household size, among others (8–11). At the onset of the COVID-19 pandemic, an important metric that also showed a positive correlation with the reproduction number of SARS-CoV-2 was the Gross Domestic Product (GDP) per capita (12–14). This suggests that the virus spread more rapidly in affluent countries due to a large percentage of the population living in densely populated cities and the significant inflow of air traffic facilitating the initial importation of a large number of cases. However, the data on this correlation was inconclusive in the subsequent phases of the COVID-19 pandemic.

Despite the implementation of measures to mitigate the spread of the virus, there are significant disparities between countries in their ability to contain the pandemic. While wealthier countries have been able to enforce stay-at-home policies by taking the appropriate measures and ensuring the safety of the most vulnerable populations, lower income countries are unable to prevent the loss of income and jobs, leading to food insecurity and reduced access to healthcare. As a result, even with mobility restrictions in place, people in these countries are still exposed to the risk of the pandemic due to the need to work and maintain their income.

The goal of this study is to highlight the connection between the different efficacy of lockdown policies observed across countries and their socioeconomic features. To this aim, we will attempt to measure the *social permeability* of these nations, which accounts for the inability of the population to remain in confinement and thus continuing social mixing that allows the disease to spread. The study of this variable allow us to distinguish unique scenarios that appear for each studied country and contribute to the already existing efforts (7) to show the varied relationships between the spread of epidemics and economic indicators.

## 2. Methods

### 2.1. Modeling

#### 2.1.1. Discrete time compartmental model

The main core of this study is the development of a compartmental model to capture COVID-19 epidemic trajectories and how they were impacted by non-pharmaceutical interventions implemented in various countries. To this aim, the model should be sufficiently complex to provide an accurate representation of the epidemic process and the primary mechanisms behind virus transmission, yet simple and adaptable enough to be applied to different countries.

**Figure 1:**
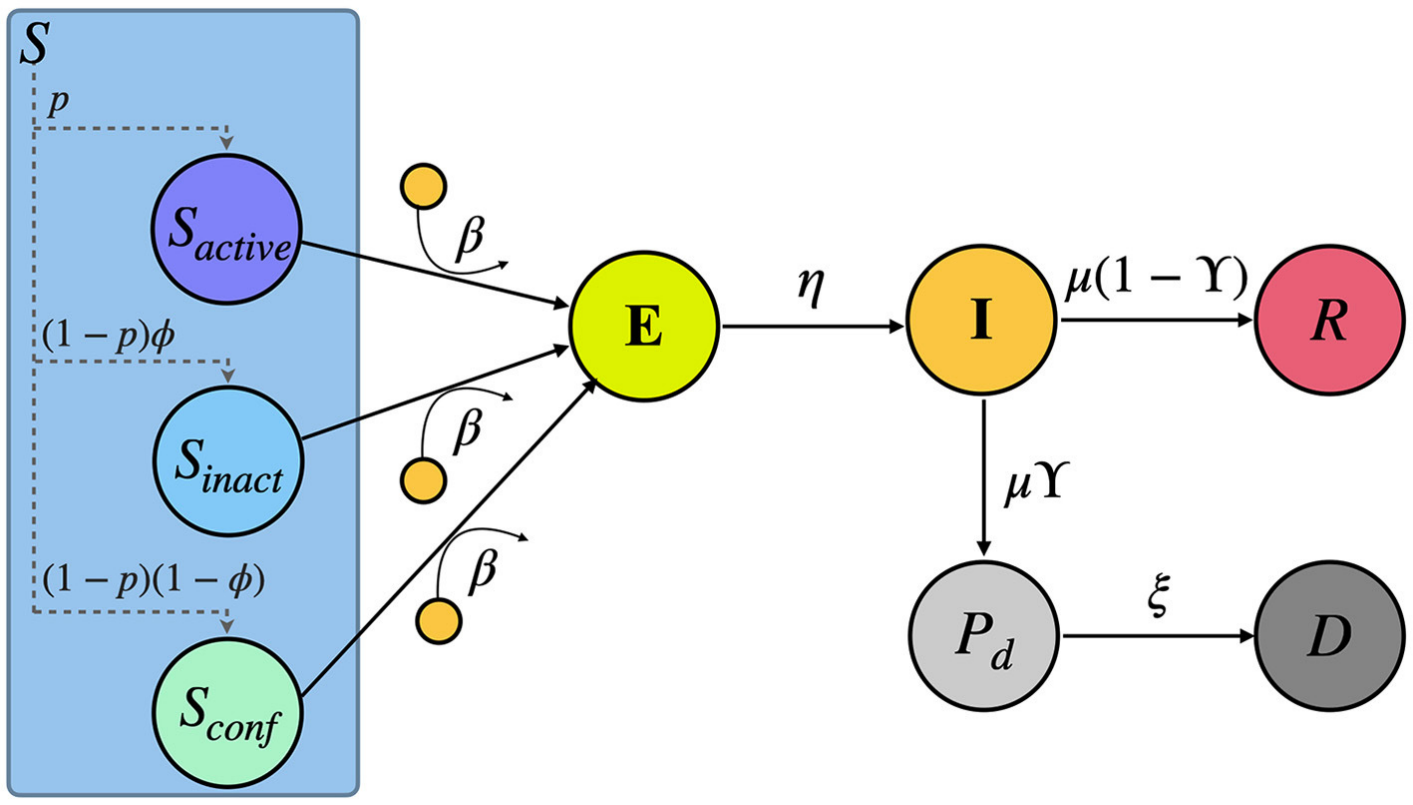
Scheme of the compartmental model here proposed. The model comprises six compartments: *Susceptible* *S*, *Exposed* *E*, *Infectious* *I*, *Recovered* *R*, *Pre-deceased* *P*_*d*_ and *Deceased* *D*. Note that, as a result of the non-pharmaceutical interventions, the *Susceptible* compartment is divided into three sub-compartments: *S*_*active*_, *S*_*inactive*_, and *S*_*confined*_ representing a fraction *p*(*t*), (1−*p*(*t*))ϕ and (1−*p*(*t*))(1−ϕ) of the total number of *Susceptible* individuals, respectively. A detailed explanation of the flows connecting these compartments can be found in the Section 2.

The proposed compartmental framework is an extended version of the *Susceptible-Exposed-Infected-Recovered* (SEIR) model (15), which allows for monitoring both the number of deaths over time for each country and the effects of non-pharmaceutical interventions. Our framework is a discrete-time model, being each time step a day. The model consists of six compartments: *Susceptible* (*S*), *Exposed* (*E*), *Infectious* (*I*), *Recovered* (*R*), *Pre-deceased* (*P*_*d*_), and *Deceased* (*D*). The flows diagram connecting these compartments is represented in Figure 1. This diagram depicts the following sequence of events: In the absence of interventions, *Susceptible* individuals (*S*) have a likelihood of contracting the virus (β) for each contact with an infected person, leading them to move to the *Exposed* compartment (*E*), meaning that they are carriers of the virus but not yet contagious. Once in compartment *E*, individuals can move to the *Infectious* compartment (*I*) with a probability η, where they can infect *Susceptible* individuals. *Infectious* individuals leave their compartment with probability μ, either recovering and entering the *Recovered* compartment (*R*) with a probability of 1−ϒ, or dying due to the disease with a probability of ϒ. In the latter case, they enter in the *Pre-deceased* compartment (*P*_*d*_) and, eventually, move to the *Deceased* compartment (*D*) with probability ξ.

Following the previous assumptions for the compartmental model, we can propose the dynamical equations driving the evolution of the individuals. In particular, the evolution from the *Infectious* stage can be straightforwardly derived, yielding:


(1) I(t+1) =ηE(t)+(1−μ)I(t) ,

(2) R(t+1) =μ(1−Υ)I(t)+R(t) ,

(3) $$P_{d}(t+1)\;=\mu \Upsilon I(t)+(1-\xi )P_{d}(t)\;,$$

(4) $$D(t+1)\;=\xi P_{d}(t)+D(t)\;.$$

The remaining equations of the model account for the contagion of the *Susceptible* individuals and are shaped by the non-pharmaceutical interventions, which manifest in a reduction in mobility and the formation of *social bubbles* throughout the populations. The impact of non-pharmaceutical interventions will be encapsulated in a fraction of the *Susceptible* population gathering the number of individuals staying at their households, not being reachable by their infectious counterparts. To model the evolution of this confined population, we assume that mobility is governed by a time-dependent parameter *p*_*act*_(*t*). In particular, for each day, a fraction *p*_*act*_(*t*) of the *Susceptible* compartment remains active (*S*_*active*_) while the rest, a fraction (1−*p*_*act*_(*t*)) of the pool of *Susceptibles*, reduce their mobility and social interactions. Of these individuals, a fraction (1−ϕ) stays at home and forms a social bubble with the rest of the members of the household (*S*_*confined*_). The rest of the individuals that became inactive but did not successfully isolate themselves completely (*S*_*inactive*_) mix with members of other households due to their *social permeability*. These three fractions of the *Susceptible* compartment can be represented as:


(5) $$\begin{array}{lll} S_{active}\left(t\right) & = & S\left(t\right)p_{act}\left(t\right) \end{array}$$

(6) $$\begin{array}{lll} S_{inactive}\left(t\right) & = & S\left(t\right)\left(1-p_{act}\left(t\right)\right)\phi \end{array}$$

(7) $$\begin{array}{lll} S_{confined}\left(t\right) & = & S\left(t\right)\left(1-p_{act}\left(t\right)\right)\left(1-\phi \right) \end{array}$$

Taking into account the aforementioned policies and every group in the *Susceptible* population, the equation governing the time evolution of the occupation of the *Exposed* compartment reads:


(8) $$\begin{array}{l} E(t+1)=S_{active}(t)P_{active}(t)+S_{inactive}(t)P_{inactive}(t)\\ \;+S_{confined}(t)P_{confined}(t)+(1-\eta )E(t)\;, \end{array}$$

where *P*_*active*_(*t*), *P*_*inactive*_(*t*) and *P*_*confined*_(*t*) account for the probability that *Susceptible* individuals belonging to each of these groups contract the disease at time *t*. We assume that the number of contacts made by each group (*k*_*active*_(*t*) and *k*_*inactive*_(*t*)) depends on the mobility levels in their respective settings, yielding:


(9) $$\begin{array}{lll} k_{active}\left(t\right) & = & \langle k_{active}\rangle p_{act}\left(t\right), \end{array}$$

(10) $$\begin{array}{lll} k_{inactive}\left(t\right) & = & \langle k_{inactive}\rangle p_{res}\left(t\right), \end{array}$$

where *p*_*act*_(*t*) represents the observed mobility of the people that travel to their daily destinations and *p*_*res*_(*t*) is the mobility of those who remain inactive in their residential areas. Likewise, 〈*k*_*active*_〉 corresponds to all contacts made by individuals in the baseline scenario whereas 〈*k*_*inactive*_〉 constitutes their interactions at home. Both can be estimated from social data existing in the literature (16).

Assuming a well-mixed population, the probabilities of contracting the disease can be calculated as:


(11) $$\begin{array}{lll} P_{active}\left(t\right) & = & 1-{\left(1-\beta \frac{I\left(t\right)}{N}\right)}^{k_{active}\left(t\right)}, \end{array}$$

(12) $$\begin{array}{lll} P_{inactive}\left(t\right) & = & 1-{\left(1-\beta \frac{I\left(t\right)}{N}\right)}^{k_{inactive}\left(t\right)}, \end{array}$$

for the active and inactive population. In addition, there is a chance that confined individuals contract the disease from other infectious members of their social bubble. These agents make *k*_*inactive*_ contacts with others in their own household. The probability of getting infected for this group of individuals depends on the number of infected people in their households as:


(13) $$\begin{array}{l} P_{confined}\left(t\right)=1-\overset{\sigma -1}{\underset{i=0}{\sum }}p\left(i\right){\left(1-\beta \frac{i}{\sigma -1}\right)}^{k_{inactive}\left(t\right)}, \end{array}$$

where σ is the number of people in each household (meaning that a susceptible member is able to make contacts with the other σ−1 residents) and the probability of finding *i* infected individuals in a household is


(14) $$p(i)=\left(\begin{array}{c} \sigma -1\\ i \end{array}\right){\left(\frac{I(t)}{N}\right)}^{i}{\left(1-\frac{I(t)}{N}\right)}^{\sigma -1-i}\;.$$

To round off, we assume a closed population so that the occupation of the *S* compartment changes with:


(15) $$\begin{array}{l} S\left(t+1\right)=N-E\left(t+1\right)-I\left(t+1\right)-R\left(t+1\right)-P_{d}\left(t+1\right)-D\left(t+1\right). \end{array}$$

### 2.2. Data sources

#### 2.2.1. COVID-19 deaths

To calibrate our model, we rely on data regarding the daily number of fatalities in each country. As consistency across populations is crucial, the number of detected cases is not a suitable metric due to surveillance issues that affect countries differently (17, 18). There are several contributing factors to the possible discrepancies on the reported cases. Firstly, testing strategies can vary across populations, with some focusing on high-risk groups or areas, while not accounting for asymptomatic cases. Additionally, limited testing capacity can lead to an underestimation of the true number of infections, particularly in areas with high community transmission. Furthermore, differences in the definition and reporting of cases, as well as demographic variations such as age, gender, and underlying health conditions, can make it challenging to compare trends between different countries or regions.

The accuracy of reported deaths is also not guaranteed for similar reasons, including possible changes in definition and reporting delays. To address these reporting issues, we specifically choose countries with continuous and consistent records of this information, which are listed in Table 1. The selection of the countries was performed according to two criteria: there must be a peak of at least 10 deaths, and the daily mobility and number of deaths have to be available and consistent for this entire duration. The data used in this study is extracted from the official daily counts of COVID-19 deaths reported for countries by the World Health Organization and smoothed using a 7-day rolling average.

**Table 1:** Average contacts of active, 〈*k*_*active*_〉, and inactive, 〈*k*_*inactive*_〉, average household size σ, GDP per capita and minimum level of mobility during lockdown for each country.

**Country**	**Country code**	**〈*k*_*active*_〉**	**〈*k*_*inactive*_〉**	**σ**	**GDP per capita (US$)**	***p*_*min*_**
Argentina	AR	14.12	3.84	2.95	8475.73	0.15
Austria	AT	12.48	3.13	2.27	48105.63	0.17
Bangladesh	BD	16.34	3.55	4.26	2000.64	0.25
Belgium	BE	11.38	2.88	2.36	45028.32	0.21
Bolivia (Plurinational State of)	BO	17.04	3.03	3.53	3133.1	0.12
Bulgaria	BG	12.73	4.06	2.34	10058.08	0.35
Canada	CA	12.57	3.1	2.45	43559.71	0.43
Chile	CL	13.71	3.66	3.04	13231.71	0.31
Colombia	CO	15.26	3.6	3.53	5332.77	0.18
Egypt	EG	15.67	3.64	4.13	3608.84	0.38
France	FR	11.78	3.1	2.22	38958.6	0.12
Germany	DE	6.86	1.79	2.05	45908.72	0.38
Greece	GR	11.79	3.18	2.44	18117.07	0.21
Guatemala	GT	18.98	4.08	4.81	4331.69	0.31
Honduras	HN	18.27	4.23	3.87	2405.73	0.17
Hungary	HU	12.07	3.46	2.6	16128.65	0.39
Indonesia	ID	15.26	3.14	3.86	3869.59	0.54
Iraq	IQ	20.64	4.35	6.35	4145.86	0.33
Ireland	IE	12.47	3.43	2.83	86250.99	0.15
Israel	IL	13.6	3.84	3.14	47033.59	0.14
Italy	IT	14.37	2.94	2.4	31238.05	0.11
Kuwait	KW	16.42	4.16	5.8	24809.04	0.12
Luxembourg	LU	16.37	3.46	2.41	117181.7	0.17
Malaysia	MY	15.4	3.59	4.56	10401.79	0.2
Mexico	MX	15.42	4.04	3.75	8325.57	0.4
Morocco	MA	14.3	3.72	4.58	3108.18	0.18
Nigeria	NG	20.47	4.16	4.66	2085.47	0.44
Pakistan	PK	18.65	4.18	6.8	1167.22	0.31
Panama	PA	14.62	3.45	3.64	12269.05	0.14
Philippines	PH	17.04	3.72	4.23	3298.83	0.17
Poland	PL	13.93	4.42	2.81	15764.11	0.32
Portugal	PT	11.88	3.03	2.66	22413.04	0.23
Romania	RO	11.97	3.41	2.88	12928.58	0.27
Russian Federation	RU	12.88	3.42	2.58	10165.51	0.45
Saudi Arabia	SA	15.64	4.1	5.6	20110.32	0.27
South Africa	ZA	15.92	3.94	3.36	5094.38	0.24
Spain	ES	12.02	3.19	2.69	27408.63	0.08
Switzerland	CH	13.14	3.11	2.21	86918.65	0.19
Turkiye	TR	13.72	3.72	4.07	8538.13	0.27
Ukraine	UA	12.65	3.48	2.53	3557.48	0.47
The United Kingdom	GB	9.48	2.25	2.27	40718.22	0.22
United States of America	US	12.6	3.24	2.49	63122.59	0.54

#### 2.2.2. Mobility reduction data

We extract the level of mobility inside each country at a certain time, *p*_*act*_(*t*) and *p*_*res*_(*t*), from the Google COVID-19 Community Mobility Reports. Among the different types of movements included in this study, we focus on the *Retail and Recreation* category for the active individuals and the *Residential* category for the inactive ones. For each day, the level of mobility is computed by comparing the amount of flows recorded for this day with their median values measured in a pre-pandemic baseline scenario, spanning 5 weeks from January 3 to February 6, 2020. To reduce data noise, we smooth the curves using a 7-day rolling average.

#### 2.2.3. Socioeconomic data

A key component of the epidemiological model is the average number of people an individual encounters (contacts) throughout their day, which varies from one country to another and has been measured in a certain number of them taking into account the heterogeneities in the populations. The dataset used for the simulation comes from a study (16) where the authors extrapolate the known data to 152 countries and provide contact matrices representing the number of contacts a person of each age group has with the others in different settings. For each country, we obtain the average number of contacts from an active individual 〈*k*_*active*_〉 as the weighted average of the total number of contacts made by each individual of each group in all the settings, taking into account the population age pyramid of this country (19). To compute the same quantity for inactive (controlled) individuals 〈*k*_*inactive*_〉, we repeat the same process by just accounting for the contacts made at home.

The average number of residents in a single home is also an important parameter of the model, and was reported by the United Nations (20) for most countries in the world. The household size σ is available at https://www.un.org/development/desa/pd/data/household-size-and-composition, and in the model has been rounded to the nearest integer in order to follow the equations.

Lastly, the Gross Domestic Product (GDP) per capita is taking into account to find a relation between the wealth of different populations and the success of their confinement policies. It is available at www.worldbank.org.

These country-dependent parameters (〈*k*_*active*_〉, 〈*k*_*inactive*_〉 and the GDP per capita) are summarized in Table 1.

#### 2.2.4. Epidemiological parameters

Some of the parameters in relation to the compartmental model have already been determined and are fixed based on the literature:

- η: Probability of leaving the *E* compartment. It is related to the average duration of the incubation period. We fix its value to η = 1.0/5.2 (21).
- μ: Probability of leaving the *I* compartment. It is related to the average duration of the infectious windows after contracting and incubating the virus. We fix its value to μ = 1.0/4.2 (21).
- ϒ: Infection fatality rate which, as reported in (22) and (23), is estimated to be ϒ = 0.01.

### 2.3. Model calibration

#### 2.3.1. Approximate Bayesian Computation (ABC)

The Approximate Bayesian Computation (ABC) method, as described in (24) and (25), provides a solution to Bayesian inference problems where computing the likelihood function and its further exploration becomes cumbersome. ABC works by generating synthetic trajectories using a set of parameters and then accepting or rejecting those parameters based on how well the synthetic trajectories match real data. This approach allows the construction of approximate posterior distributions.

There are several ways of exploring the posterior distribution of the parameters, one of the simplest being the ABC rejection algorithm (26), which is used in our case. To quantify the goodness of a given trajectory generated by a set of parameters $\vec{\theta }$, we use a logarithmic distance function $\rho \left(\vec{\theta }\right)$ defined as:


(16) $$\begin{array}{l} \rho \left(\vec{\theta }\right)\equiv \underset{t}{\sum }\log\left[|D_{obs}\left(t\right)-D_{\vec{\theta }}\left(t\right)|+1\right], \end{array}$$

where *D*_*obs*_(*t*) represents the observed daily fatalities at time *t* and $D_{\vec{\theta }}\left(t\right)$ its value predicted by the synthetic trajectory. Note that, among all possible choices for this goodness function, we have chosen a logarithmic function not to under-represent the initial stage of the epidemics, where there are fewer deaths.

The ABC rejection algorithm builds the posterior distribution for the model parameters by sampling them from the trajectories fulfilling that $\rho \left(\vec{\theta }\right)<\epsilon$, where ϵ is a tolerance threshold. In our case, we run two rounds of the ABC rejection algorithm. In the first round, we draw 20·10^6^ random samples of variables from the prior distributions and compute the distance between the synthetic trajectories and the real data by taking into account the period between February 20 and May 20, 2020. We set a dynamical threshold ϵ_*i*_ to accept those 1, 000 trajectories providing the best fits for the data in each country. We construct the prior distributions for the second from the accepted trajectories in the first one and the process is repeated. This second iteration allows the algorithm to give more accurate results for each country due to the intrinsic variability of the parameters across countries.

#### 2.3.2. Model free parameters

The numerical iteration of Equation (1) allows one to obtain synthetic trajectories capturing the evolution of individuals in each of the compartments. The parameters of the model that are not fixed from the literature will be left for calibration via the ABC method. These parameters are:

- β: This parameter represents the probability of infection, which varies from one country to another due to factors such as population density, urbanization, and use of masks. The prior distribution of this parameter is β~*U*(0.01, 0.3).
- ϕ: The permeability of the confinement, which is the main objective of the study to fit. A low permeability means a high effectiveness of the non-pharmaceutical policies due to a good compliance from the population. The prior distribution of this parameter is ϕ~*U*(0, 1).
- ξ: The probability of leaving the *P*_*d*_ compartment to die because of the disease. The prior distribution of this parameter is ξ~*U*(1/18, 1/6).
- *T*: The estimated number of days that have elapsed since the first case of the disease in the country and the day chosen as the starting point for comparison with observed deaths, which for all countries corresponds to 2020-02-20. The prior distribution of this parameter reads *T*~*U*(0, 100).
- δ: The delay in death notification. The prior distribution of this parameter is set at δ~*U*(2, 20).

Note that δ is not a strictly needed parameter to run the model but becomes essential to make synthetic trajectories compatible with real data (27). In all the cases, the prior distributions chosen are broad enough to avoid biasing the inference of the posterior distributions.

### 2.4. Relationship between GDP per capita and countries permeability

In this section, we explain how we link the inferred permeability distributions of individual countries with certain economic indicators, such as GDP per capita. To obtain a meaningful association, we should exclude countries for which the model does not provide a reasonable fit, as well as those where mobility limitations did not substantially impact the control of the epidemic.

On the one hand, to determine which countries have been correctly modeled, we calculate the relative area between the estimated curve and the real data using the following equation:


(17) $$\varepsilon (\vec{\theta })=\frac{\sum_{t}\left|D_{obs}(t)-D_{\vec{\theta }}(t)\right|}{\sum_{t}D_{obs}(t)}\;.$$

For the subsequent analysis, we discard those countries for which $\min\left(\varepsilon \left(\vec{\theta }\right)\right)>0.4$ as the model does not fit well the epidemic trajectories there.

Once these countries are discarded, we quantify the relationship between permeability and GDP per capita by performing a non-linear regression fitting the permeability to the following function:


(18) $$\begin{array}{l} \phi \left(x\right)=ax^{-b} \end{array}$$

where *x* is the GDP per capita. As our information about permeability comes from posterior distributions, we conduct 1, 000 independent fits by sampling diverse sets of permeability values from these distributions. The confidence interval of the regression curve is calculated as the percentile 2.5 to the percentile 97.5 of the individuals fits obtained.

## 3. Results

**Figure 2:**
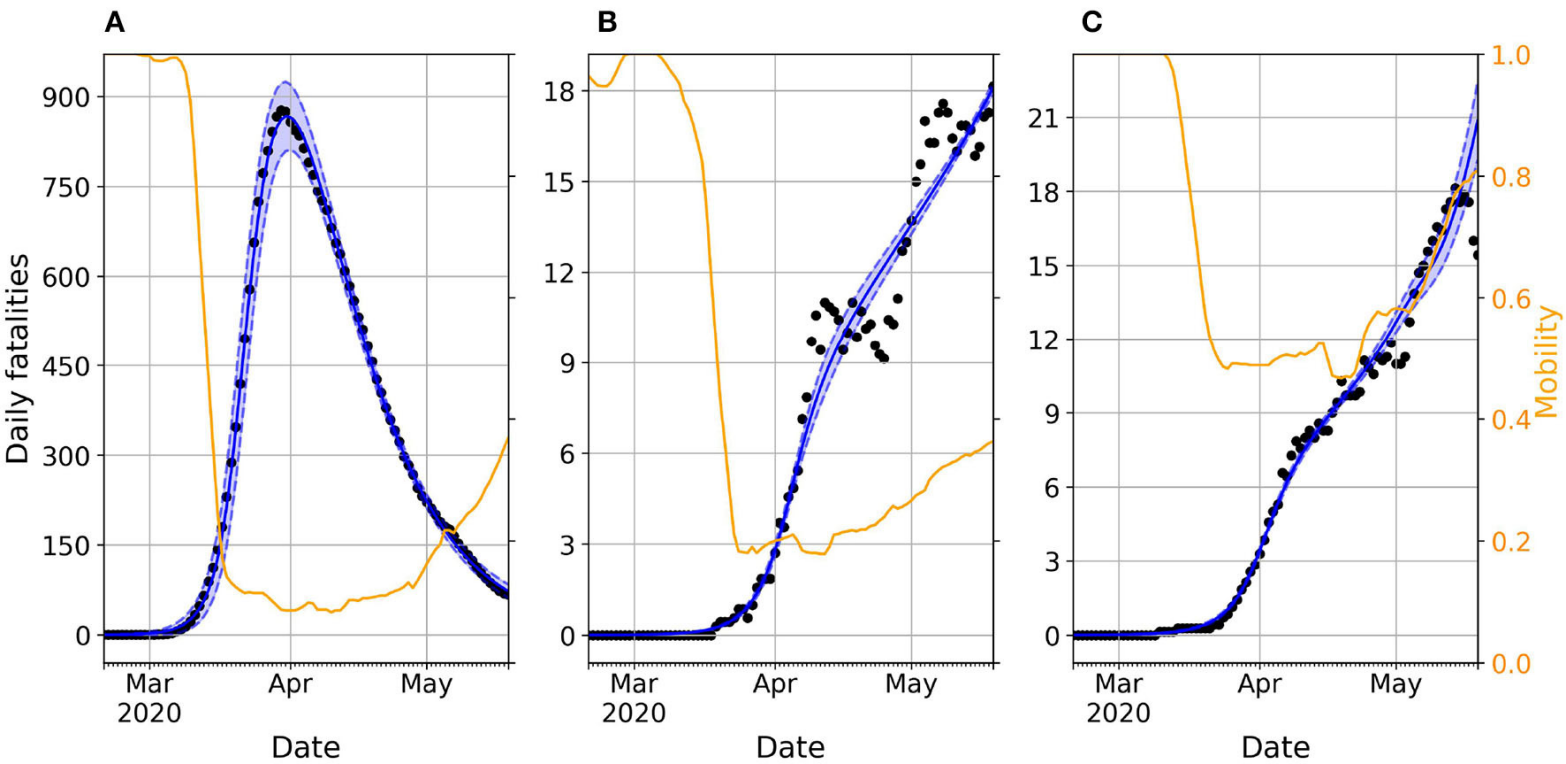
Daily evolution of the number of deaths in Spain **(A)**, Colombia **(B)**, and Ukraine **(C)**. In all the panels, dots represent real reported data whereas the blue shadowed region corresponds to the 95% prediction interval of the accepted trajectories after calibrating the model. The blue solid line represents the median trajectory whereas the orange line corresponds to the time variation of mobility compared with a baseline pre-pandemic scenario spanning from January 3 to February 6, 2020.

We calibrate our model to real data using the Approximate Bayesian Computation (ABC) scheme described in the Methods section. The results of the calibration for each considered country can be found in Supplementary Figure 1. One important finding is that, despite its simplicity, our simple model accurately captures the time evolution of reported deaths for most countries and confirms the assumption that the mobility reduction has a direct effect on the number of daily contagions, which decreases as stricter policies are put in place. Figure 2 illustrates this by showing the real and simulated epidemic trajectories for Spain, Colombia, and Ukraine. The selected countries represent three distinct types of behavior observed in our study. While Spain and Colombia implemented similar lockdown policies resulting in comparable reductions in average mobility, their outcomes were vastly different: Spain managed to stop the spread and bend the curve, whereas Colombia experienced a steady growth in casualties. This pattern of steady increase is also observed in Ukraine, which had a milder reduction in mobility compared to Spain and Colombia.

The unequal impact of mobility reduction on epidemic containment can be captured by the social permeability ϕ parameter in our model, which modulates the effective reproductive number of the circulating virus, as explained in (28). Namely, low permeability values significantly reduce the pool of susceptible individuals exposed to the virus due to the lower household mixing, whereas high permeability values means that all the individuals remain vulnerable to the virus but with a reduced exposure due to their hampered social activity.

**Figure 3:**
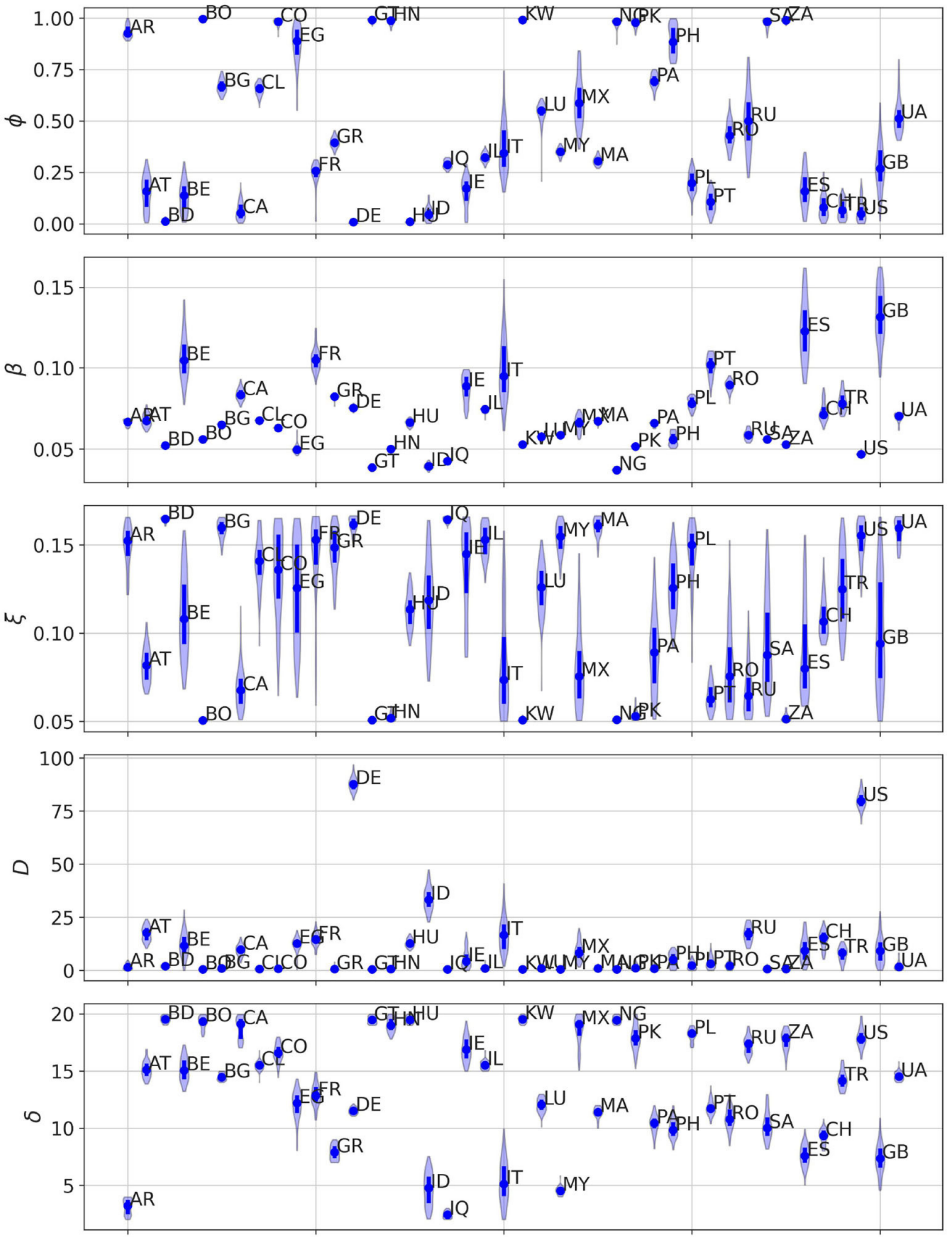
Posterior distribution for each of the free parameters of our model (*ϕ*, *β*, ξ, *T*, *δ*) obtained after calibrating the model in each of the countries here analyzed. For each parameter, dots denote the median value of the distribution whereas the solid line represents the IQR of each distribution.

In Figure 3, we present the posterior distributions obtained of social permeability for each country analyzed in this study. Focusing on the specific case of Colombia and Spain, we confirm that lower efficiency of mobility reductions in Colombia translates to higher permeability values compared to those inferred for Spain. While not the focus of our manuscript, other model parameters also provide insightful information about the impact of the first COVID-19 epidemic wave and the associated contention measures across countries. For instance, the inferred values of parameter *T* enable to reconstruct the time of onset of the outbreak in each country, whereas parameter δ accounts for the heterogeneous delay in reporting deaths. Nonetheless, conclusions on these parameters should be drawn with caution because of the correlations between their posterior distributions, as illustrated in Supplementary Figures 2–4 for the case of Spain, Colombia and Ukraine, respectively.

To round off, we check whether we can connect the heterogeneous permeability values inferred for each country with their corresponding socioeconomic features. In order to establish a meaningful link, we narrow our focus to countries where the model accurately predicts the course of the disease. We determine this accuracy by calculating the normalized distance $\varepsilon \left(\vec{\theta }\right)$ between the data and the model trajectories, which enables us to establish a threshold and exclude countries where the model does not perform well. This procedure is described in more detail in the Section 2 and the distribution of the minimum normalized distances ε^min^ observed across countries is represented in Supplementary Figure 5.

**Figure 4:**
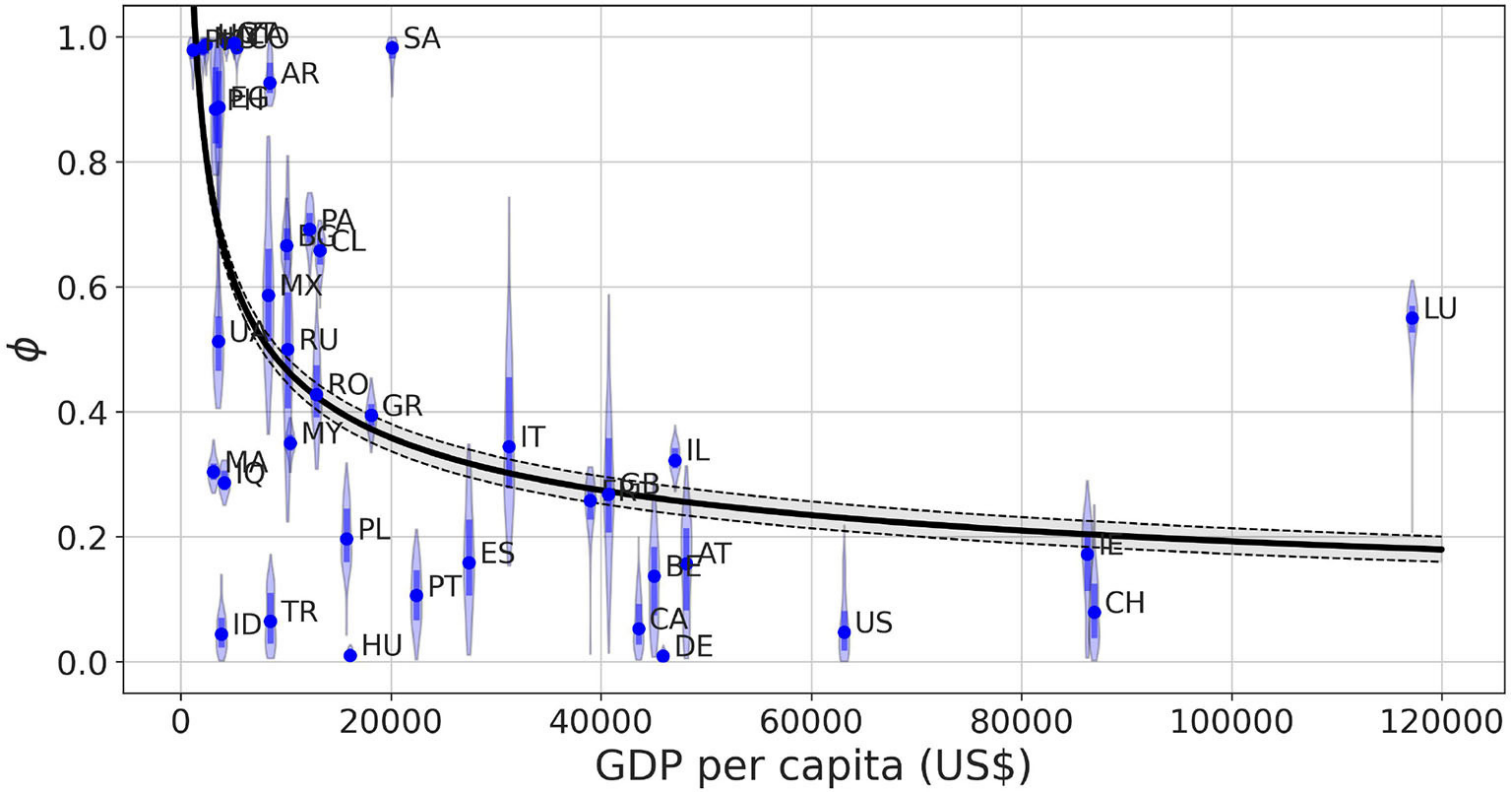
Posterior distribution obtained for the permeability parameter *ϕ* as a function of the GDP per capita of the country in which the model is calibrated. The shadowed region of the fit shows the 95% prediction interval of the trajectories obtained via non-linear regression *ϕ*(*x*) = *ax*^−*b*^, where *x* stands for GDP per capita and the parameters result in *a* = 16 ± 4.12, *b* = 0.39 ± 0.03. The solid line represents the average value of the fitted trajectories for each *x*. The Spearman correlation coefficient ρ_*S*_ between both variables is ρ_*S*_ = −0.590 with *p* < 10^−4^.

Figure 4 represents the posterior distribution of the social permeability against the gross domestic product (GDP) per capita of the selected countries. The tendency showcased in the figure indicates that there is in fact a negative statistically significant correlation between the wealth of a country and the ability of its inhabitants to properly follow the restrictions and stay in lockdown. This negative correlation between the permeability and GDP per capita is further supported by the non-linear regression of the data described in the Section 2.

For the sake of completeness, we study the influence of possible confounding factors on this correlation such as mobility reduction and deaths caused by the disease. Supplementary Figure 6 shows that the permeability values have no correlation with the minimum observed mobility for each one of them, meaning the model can separate the level of mobility reduction and the effectiveness of the confinement without one depending on the other. Regarding the relationship between permeability and death toll per capita in each country, we observe in Supplementary Figure 7 low permeability values for those countries with higher number of fatalities but a large variability without any clear relationship in those less severely affected.

## 4. Discussion

The COVID-19 pandemic has had an undeniable impact on the world and has exposed the existing social and economic inequalities within many countries (29, 30). To address this issue, various countries have implemented policies and non-pharmaceutical interventions to control the virus's spread and reduce the number of casualties. However, the pandemic's impact has not been evenly distributed across society, with certain groups suffering more severe consequences than others (31, 32). This inequality is not limited to individual countries, but also occurs across nations due to various challenges that low income countries face in implementing measures to prevent transmission. Low income countries encounter numerous obstacles, such as inadequate infrastructure, a lack of public trust, and a high percentage of individuals working in the informal sector, who cannot work remotely from home and lose their source of income (33–35). These factors have resulted in significant challenges in controlling the virus in many low income countries, underscoring the pressing need for a global effort to address the pandemic equitably and effectively.

The focus of our research has been to explore the impact of socioeconomic determinants on the efficacy of stay-at-home measures in controlling the spread of COVID-19. By using the change in mobility as a metric for the strictness of the restrictions, without the assumption that they are an accurate quantitative representation of the real level of confinement that the population went through, we have been able to replicate the epidemic trajectory in 42 countries. Our findings indicate that a reduction in mobility is strongly associated with a decrease in virus transmission. However, we recognize that this metric may not always be an accurate representation of the true level of confinement experienced by the population. To address this, we introduced the concept of social permeability, which was estimated using Approximate Bayesian Computation. Our results suggest that low-income countries tend to have a higher permeability, indicating that restrictions were less effective in achieving an efficient population confinement.

Finally, the framework here proposed constitutes a minimal approach to capture the evolution of COVID-19 pandemic under mobility restrictions and presents different limitations. First, the model assumes a well-mixed population inside each country, neglecting possible spatial heterogeneities existing among its different regions and resulting in the aggregated values for the variables not representing a fair indicator of the evolution of the pandemic. In this case, the aggregated values for these variables do not represent a fair indicator of the evolution of the pandemic. In addition, our model overlooks those policies which might play an important role in bending the epidemic curves while not entailing a significant reduction in the mobility levels of the population. Some examples might be social distancing of the population, prophylaxis measures such as hands hygiene or wearing masks or ban of massive gatherings of individuals. Furthermore, mobility reduction levels were obtained from the Google COVID-19 mobility dataset, which relies on the mobility patterns estimated from smartphone users that have opted in to Google's Location History feature, which is off by default. Because of this, the results are based on the assumption that these users represent the behavior of the entire population in their respective countries. Despite all these limitations, we hope that our model paves the way to the elaboration of more sophisticated frameworks addressing the relevance of the interplay socioeconomic features and mobility reductions during epidemic outbreaks.

## Data availability statement

The original contributions presented in the study are included in the article/Supplementary material, further inquiries can be directed to the corresponding author.

## Author contributions

DS-P and JG-G conceived the research project. PV and UL performed the data analysis and calibration of the compartmental model. PV, DS-P, and JG-G drafted the manuscript. All authors contributed to the study design and methodology. All authors have read and agree with the published version of the manuscript.

## References

[1] Huang C Wang Y Li X Ren L Zhao J Hu Y. Clinical features of patients infected with 2019 novel coronavirus in Wuhan, China. Lancet. (2020) 395:497–506. 10.1016/S0140-6736(20)30183-5 31986264 PMC7159299

[2] Perra N. Non-pharmaceutical interventions during the COVID-19 pandemic: a review. Phys Rep. (2021) 913:1–52. 10.1016/j.physrep.2021.02.001 33612922 PMC7881715

[3] Kapitsinis N. The underlying factors of the COVID-19 spatially uneven spread. Initial evidence from regions in nine EU countries. Region Sci Policy Pract. (2020) 12:1027–45. 10.1111/rsp3.12340

[4] Kang D Choi H Kim JH Choi J. Spatial epidemic dynamics of the COVID-19 outbreak in China. Int J Infect Dis. (2020) 94:96–102. 10.1016/j.ijid.2020.03.076 32251789 PMC7194591

[5] Raharja A Tamara A Kok LT. Association between ethnicity and severe COVID-19 disease: a systematic review and meta-analysis. J Racial Ethnic Health Disparities. (2020) 8:1563–72. 10.1007/s40615-020-00921-5 33180278 PMC7659894

[6] Magesh S John D Li WT Li Y Mattingly-App A Jain S. Disparities in COVID-19 outcomes by race, ethnicity, and socioeconomic status: a systematic-review and meta-analysis. JAMA Netw Open. (2021) 4:e2134147. 10.1001/jamanetworkopen.2021.34147 34762110 PMC8586903

[7] Benita F Rebollar-Ruelas L Gayton-Alfaro ED. What have we learned about socioeconomic inequalities in the spread of COVID-19? A systematic review. Sustain Cities Soc. (2022) 86:104158. 10.1016/j.scs.2022.104158 36060423 PMC9428120

[8] Hu H Nigmatulina K Eckhoff P. The scaling of contact rates with population density for the infectious disease models. Math Biosci. (2013) 244:125–34. 10.1016/j.mbs.2013.04.013 23665296

[9] House T Keeling MJ. Household structure and infectious disease transmission. Epidemiol Infect. (2009) 137:654–61. 10.1017/S0950268808001416 18840319 PMC2829934

[10] Liu P McQuarrie L Song Y Colijn C. Modelling the impact of household size distribution on the transmission dynamics of COVID-19. J R Soc Interface. (2021) 18:20210036. 10.1098/rsif.2021.0036 33906389 PMC8086889

[11] Esseau-Thomas C Galarraga O Khalifa S. Epidemics, pandemics and income inequality. Health Econ Rev. (2022) 12:7. 10.1186/s13561-022-00355-1 35043257 PMC8765494

[12] Libório MP Ekel PY de Abreu JF Laudares S. Factors that most expose countries to COVID-19: a composite indicators-based approach. GeoJournal. (2022) 87:5435–49. 10.1007/s10708-021-10557-5 34873361 PMC8636286

[13] Varkey RS Joy J Sarmah G Panda PK. Socioeconomic determinants of COVID-19 in Asian countries: an empirical analysis. J Public Aff. (2021) 21:e2532. 10.1002/pa.2532 33173444 PMC7645920

[14] Baser O. Population density index and its use for distribution of COVID-19: a case study using Turkish data. Health Policy. (2021) 125:148–54. 10.1016/j.healthpol.2020.10.003 33190934 PMC7550260

[15] Keeling M Rohani P. Modeling Infectious Diseases in Humans and Animals. Princeton, NJ: Princeton University Press (2008).

[16] Prem K Cook AR Jit M. Projecting social contact matrices in 152 countries using contact surveys and demographic data. PLoS Comput Biol. (2017) 13:e1005697. 10.1371/journal.pcbi.1005697 28898249 PMC5609774

[17] Reese H Iuliano AD Patel NN Garg S Kim L Silk BJ. Estimated incidence of coronavirus disease 2019 (COVID-19) illness and hospitalization–United States, February–September 2020. Clin Infect Dis. (2020) 72:e1010–7. 10.1093/cid/ciaa1780 33237993 PMC7717219

[18] Iuliano AD Chang HH Patel NN Threlkel R Kniss K Reich J. Estimating under-recognized COVID-19 deaths, United States, March 2020-May 2021 using an excess mortality modelling approach. Lancet Region Health Am. (2021) 1:100019. 10.1016/j.lana.2021.100019 34386789 PMC8275579

[19] Nations U. World Population Prospects, The 2019 Revision - Volume I: Comprehensive Tables. United Nations (2019). Available online at: https://www.un-ilibrary.org/content/books/9789210046428

[20] United Nations. Household Size and Composition Around the World. Economic and Social Affairs (2017).

[21] Lauer SA Grantz KH Bi Q Jones FK Zheng Q Meredith HR. The incubation period of coronavirus disease 2019 (COVID-19) from publicly reported confirmed cases: estimation and application. Ann Internal Med. (2020) 172:577–82. 10.7326/M20-0504 32150748 PMC7081172

[22] Brazeau NF Verity R Jenks S Fu H Whittaker C Winskill P. Estimating the COVID-19 infection fatality ratio accounting for seroreversion using statistical modelling. Commun Med. (2022) 2:54. 10.1038/s43856-022-00106-7 35603270 PMC9120146

[23] Bar-On YM Flamholz A Phillips R Milo R. Science Forum: SARS-CoV-2 (COVID-19) by the numbers. eLife. (2020) 9:e57309. 10.7554/eLife.57309 32228860 PMC7224694

[24] Sunnåker M Busetto AG Numminen E Corander J Foll M Dessimoz C. Approximate Bayesian computation. PLoS Comput Biol. (2013) 9:e1002803. 10.1371/journal.pcbi.1002803 23341757 PMC3547661

[25] Csilléry K Blum MG Gaggiotti OE François O. Approximate Bayesian computation (ABC) in practice. Trends Ecol Evol. (2010) 25:410–8. 10.1016/j.tree.2010.04.001 20488578

[26] Gelman A. A Bayesian formulation of exploratory data analysis and goodness-of-fit testing. Int Stat Rev. (2003) 71:369–82. 10.1111/j.1751-5823.2003.tb00203.x

[27] Gutierrez E Rubli A Tavares T. Delays in death reports and their implications for tracking the evolution of COVID-19. Covid Econom. (2020) 1:116–44. Available online at: https://papers.ssrn.com/sol3/papers.cfm?abstract_id=3645304

[28] Arenas A Cota W Gómez-Gardeñes J Gómez S Granell C Matamalas JT. Modeling the spatiotemporal epidemic spreading of COVID-19 and the impact of mobility and social distancing interventions. Phys Rev X. (2020) 10:041055. 10.1103/PhysRevX.10.041055

[29] Van Dorn A Cooney RE Sabin ML. COVID-19 exacerbating inequalities in the US. Lancet. (2020) 395:1243–4. 10.1016/S0140-6736(20)30893-X 32305087 PMC7162639

[30] Wachtler B Michalski N Nowossadeck E Diercke M Wahrendorf M Santos-Hövener C. Socioeconomic inequalities and COVID-19–A review of the current international literature. J Health Monitor. (2020) 5(Suppl 7):3. 10.25646/7059 35146298 PMC8734114

[31] Arceo-Gomez EO Campos-Vazquez RM Esquivel G Alcaraz E Martinez LA Lopez NG. The income gradient in COVID-19 mortality and hospitalisation: an observational study with social security administrative records in Mexico. Lancet Region Health Am. (2022) 6:100115. 10.1016/j.lana.2021.100115 34778865 PMC8578731

[32] Drefahl S Wallace M Mussino E Aradhya S Kolk M Brandén M. A population-based cohort study of socio-demographic risk factors for COVID-19 deaths in Sweden. Nat Commun. (2020) 11:1–7. 10.1038/s41467-020-18926-3 33037218 PMC7547672

[33] Lou J Shen X Niemeier D. Are stay-at-home orders more difficult to follow for low-income groups? J Transport Geogr. (2020) 89:102894. 10.1016/j.jtrangeo.2020.102894 33519126 PMC7832451

[34] Brodeur A Grigoryeva I Kattan L. Stay-at-home orders, social distancing, and trust. J Popul Econ. (2021) 34:1321–54. 10.1007/s00148-021-00848-z 34177123 PMC8214058

[35] Huang X Lu J Gao S Wang S Liu Z Wei H. Staying at home is a privilege: evidence from fine-grained mobile phone location data in the United States during the COVID-19 pandemic. Ann Am Assoc Geograph. (2022) 112:286–305. 10.1080/24694452.2021.1904819

