# Supplementary material for: Socioeconomic determinants of stay-at-home policies during the first COVID-19 wave
Source: Front Public Health. 2023 Jul 5;11:1193100. doi: 10.3389/fpubh.2023.1193100 (PMC10354257; doi:10.3389/fpubh.2023.1193100)
Supplement: Supplementary file 1 [file Data_Sheet_1.PDF]

Supplementary Material

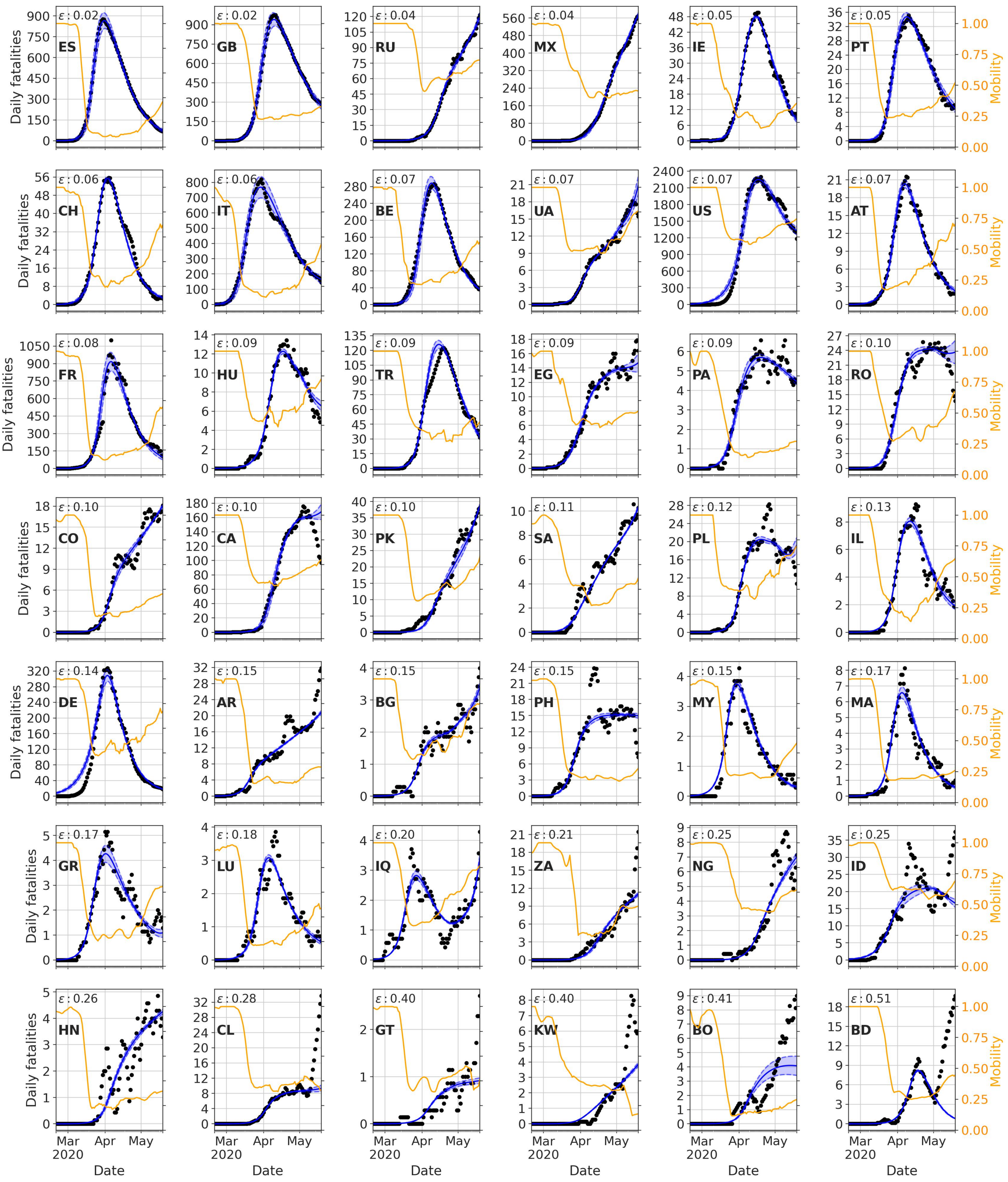

Figure S1. Daily evolution of the number of deaths in each of the 42 countries here analyzed. In all the panels, dots represent real reported data whereas the blue shadowed region corresponds to the 95% prediction interval of the accepted trajectories after calibrating the model. The blue solid line represents the median trajectory whereas the orange line corresponds to the time variation of mobility compared with a baseline pre-pandemic scenario spanning from January 3 to February 6, 2020.

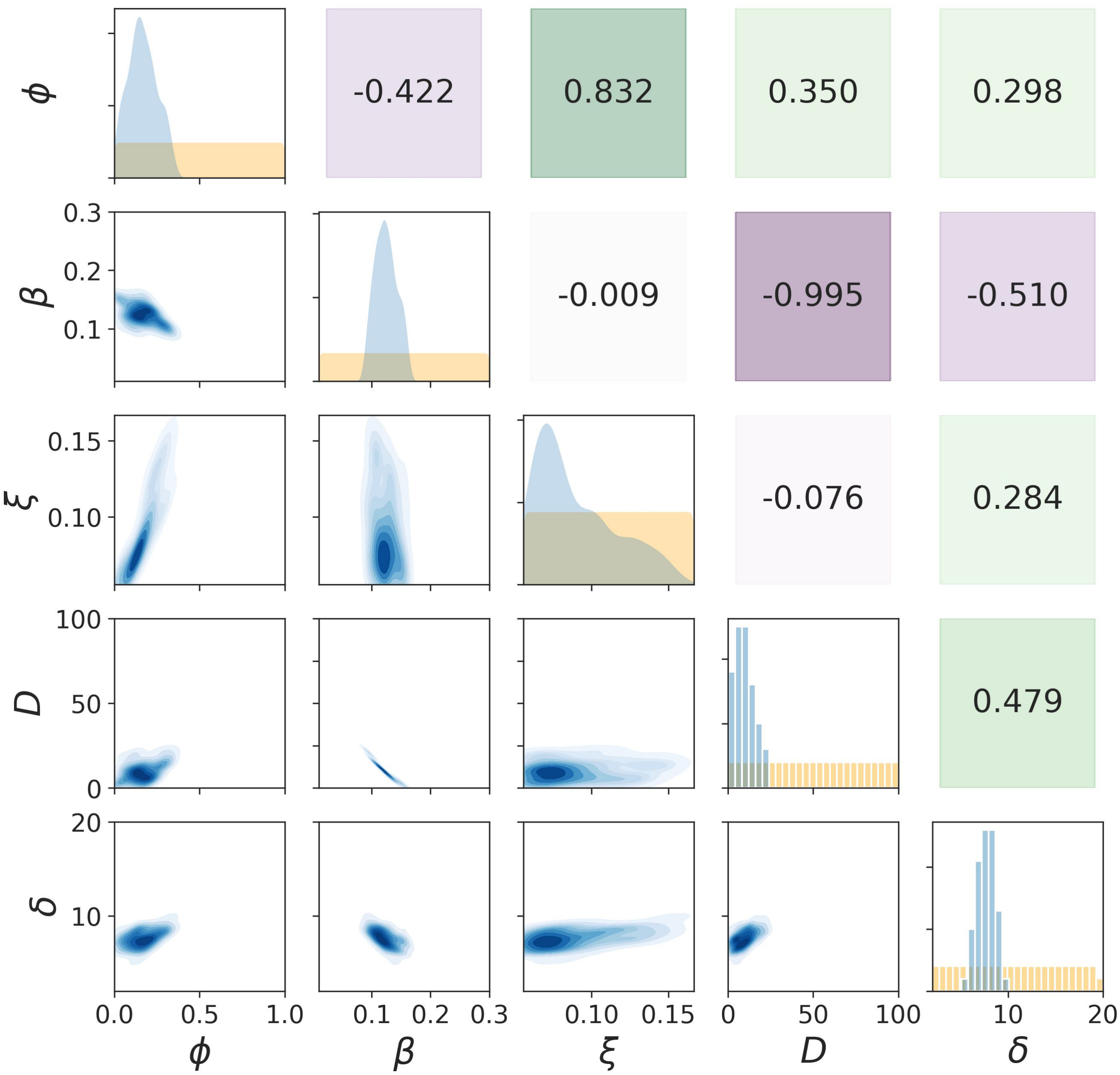

Figure S2. Model calibration in Spain. Panels in the diagonal show the prior distribution for each parameters (orange) and its posterior distribution after calibration (blue). Lower diagonal panels represent the scatter plots relating the pairwise combinations of values in the accepted trajectories whereas the upper diagonal panels contains the Spearman correlation coefficient between each pair of set of parameters found in the accepted trajectories.

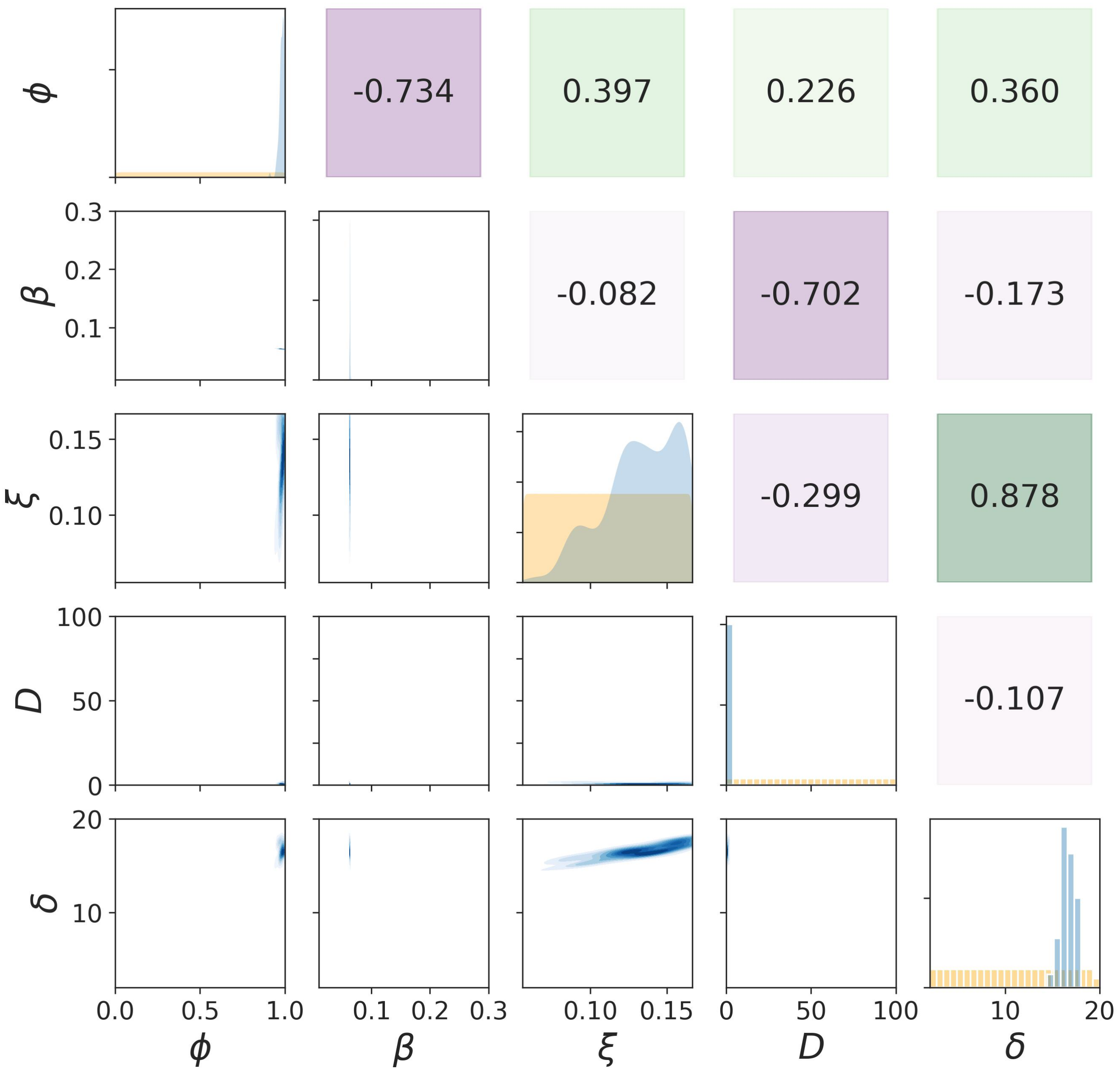

Figure S3. Model calibration in Colombia. Panels in the diagonal show the prior distribution for each parameters (orange) and its posterior distribution after calibration (blue). Lower diagonal panels represent the scatter plots relating the pairwise combinations of values in the accepted trajectories whereas the upper diagonal panels contains the Spearman correlation coefficient between each pair of set of parameters found in the accepted trajectories.

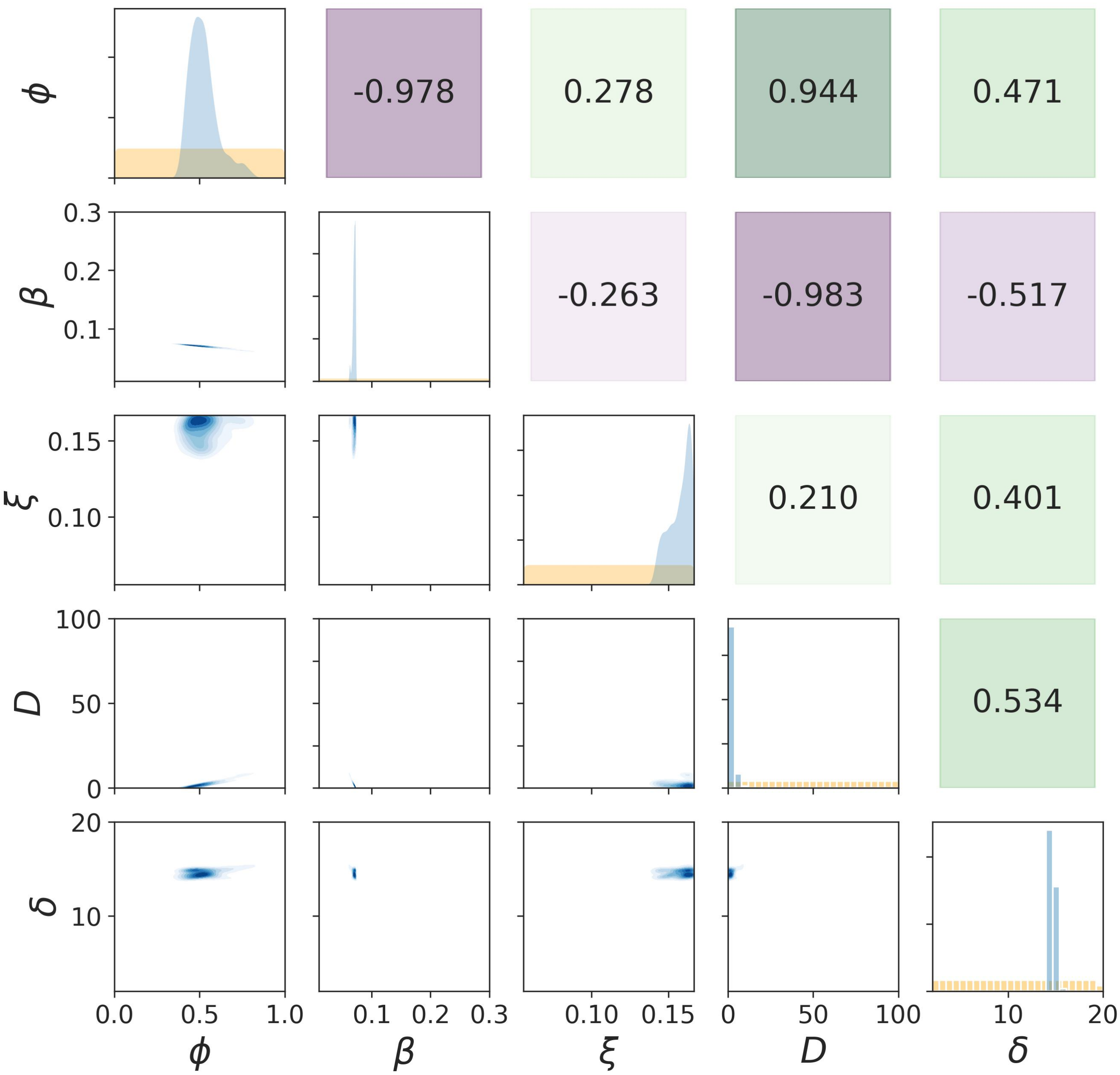

Figure S4. Model calibration in Ukraine. Panels in the diagonal show the prior distribution for each parameters (orange) and its posterior distribution after calibration (blue). Lower diagonal panels represent the scatter plots relating the pairwise combinations of values in the accepted trajectories whereas the upper diagonal panels contains the Spearman correlation coefficient between each pair of set of parameters found in the accepted trajectories.

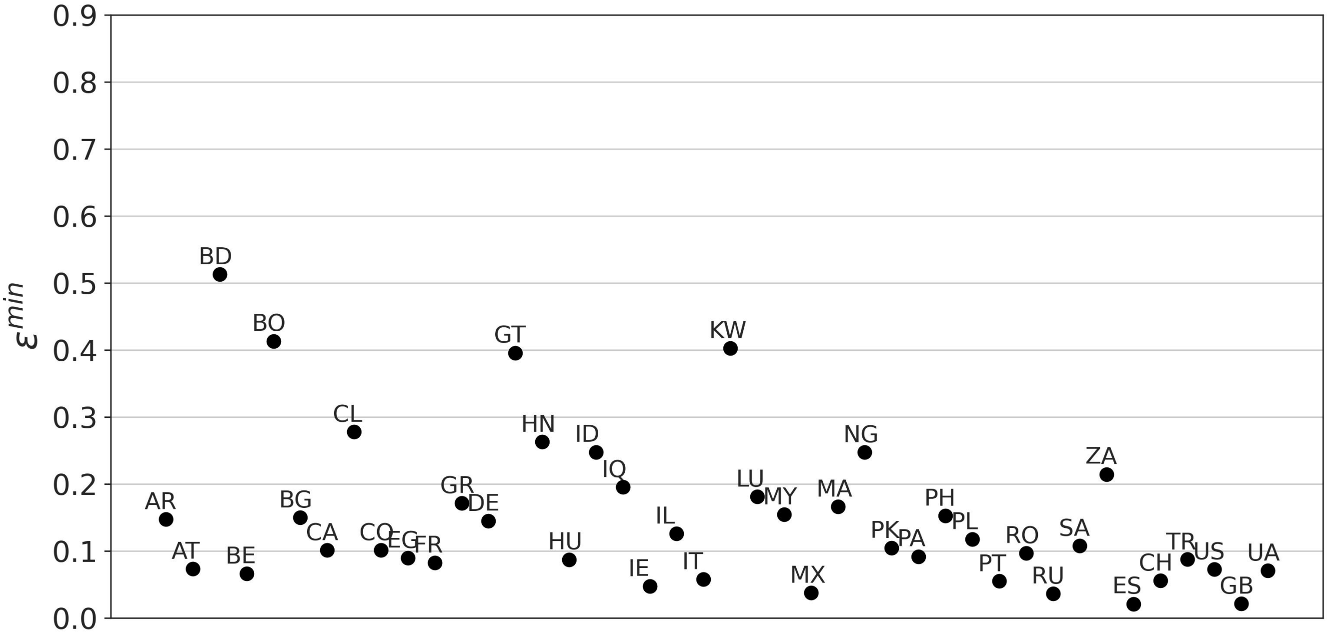

Figure S5. Minimum relative error of the fit $\varepsilon^{\mathrm{min}}$ for every country as defined in equation (13).

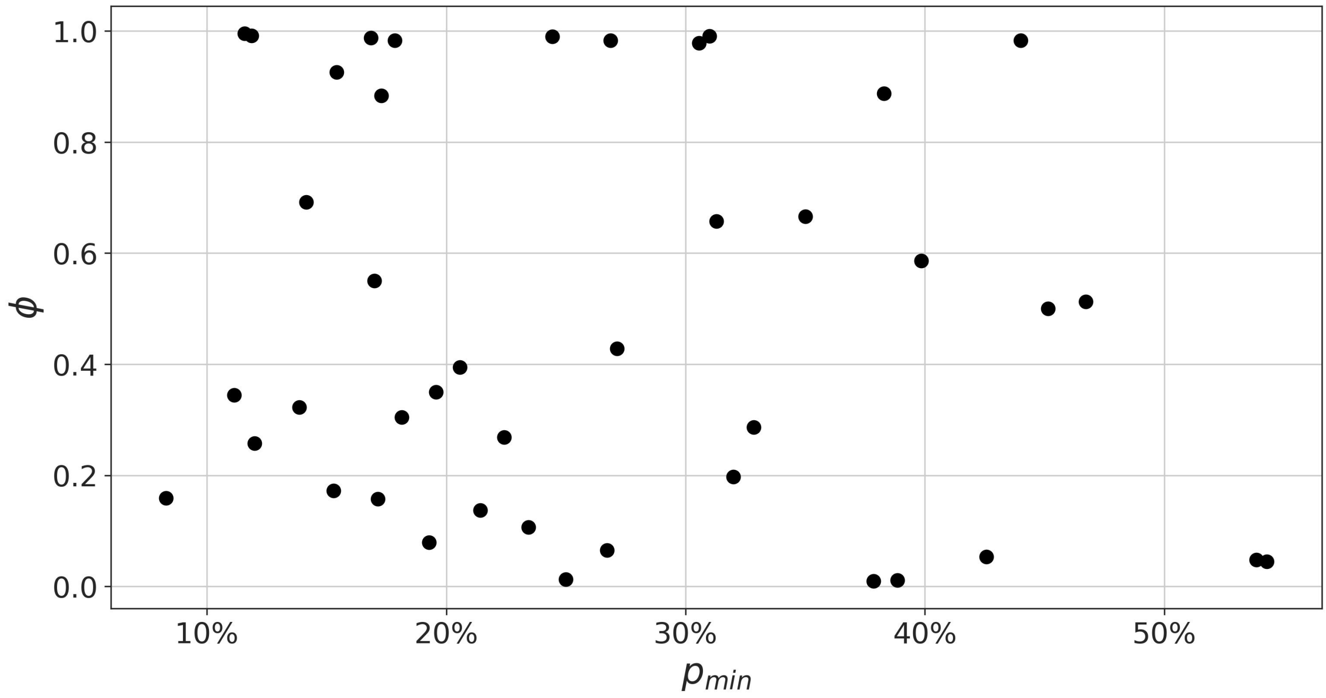

Figure S6. Median of the permeability distribution from the fit of each country as a function of the minimum mobility reached by its population.

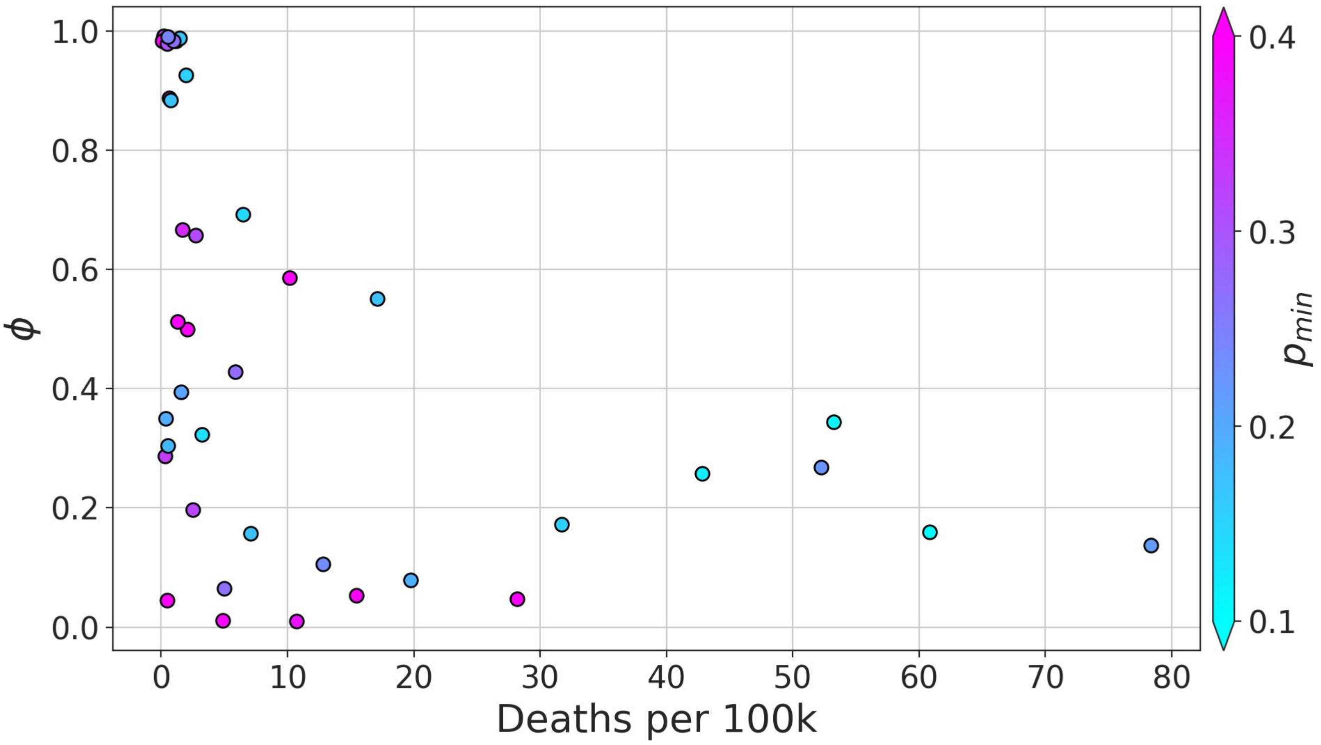

Figure S7. Median of the permeability distribution from the fit of each country as a function of the number of reported deaths during the studied period. The color code represents the minimum mobility achieved by the population of each country in relation to its original pre-pandemic value.
